# Towards Laterally Resolved Ferromagnetic Resonance with Spin-Polarized Scanning Tunneling Microscopy

**DOI:** 10.3390/nano9060827

**Published:** 2019-05-31

**Authors:** Marie Hervé, Moritz Peter, Timofey Balashov, Wulf Wulfhekel

**Affiliations:** 1 Physikalisches Institut, Karlsruhe Institute of Technology, Wolfgang-Gaede-Strasse 1, 76131 Karlsruhe, Germany; moritz.peter@gmx.de (M.P.); Timofey.Balashov@kit.edu (T.B.); wulf.wulfhekel@kit.edu (W.W.); 2 Institut des Nanosciences de Paris, Sorbonne Université and CNRS-UMR7588, 75005 Paris, France

**Keywords:** ferromagnetic resonance, spin-polarized scanning tunneling microscopy, magnetic vortices

## Abstract

We used a homodyne detection to investigate the gyration of magnetic vortex cores in Fe islands on W(110) with spin-polarized scanning tunneling microscopy at liquid helium temperatures. The technique aims at local detection of the spin precession as a function of frequency using a radio-frequency (rf) modulation of the tunneling bias voltage. The gyration was excited by the resulting spin-polarized rf current in the tunneling junction. A theoretical analysis of different contributions to the frequency-dependent signals expected in this technique is given. These include, besides the ferromagnetic resonance signal, also signals caused by the non-linearity of the I(U) characteristics. The vortex gyration was modeled with micromagnetic finite element methods using realistic parameters for the tunneling current, its spin polarization, and the island shape, and simulations were compared with the experimental results. The observed signals are presented and critically analyzed.

## 1. Introduction

Spin-polarized scanning tunneling microscopy (SP-STM) is a powerful technique to study the local magnetic structure of surfaces [1,2]. One of the limitations of this technique is its time resolution, which is limited by the bandwidth of the transimpedance amplifier used to convert the tunneling current in the pA/nA range to a manageable voltage. Typical bandwidths are around several kHz, far too low to track the magnetic precession dynamics of magnetic objects typically in the GHz domain. Some extensions to STM have been proposed to overcome this technical barrier and to achieve higher temporal resolution. In order to study dynamics in the time domain, pump-probe-based experiments have been proposed [3,4,5,6]. These experiments add fast pump and probe pulses to the tunneling bias voltage. The two pulses can be delayed with respect to each other. The first pulse (pump pulse) is used to excite the sample, and the decay of the excitation is probed by the second pulse (probe pulse). Depending on the delay, a Direct (DC) tunneling current can be detected using conventional transimpedance amplifiers. With an all-electronic pump probe experiment and 50-ns time resolution, Loth et al. [5] have successfully measured the spin relaxation of single Fe-Cu dimers on CuN thin films. Their work stimulated the area of time-resolved STM, so that other groups reported on the development of all-electronic pump probe experiments allowing even higher time resolution down to 120 ps [7]. Another approach to study the Larmor precession of single atoms on surfaces in the frequency domain has been proposed by Manassen et al. [8,9]. To split the spin states of a paramagnetic object, an out-of-plane magnetic field was applied such that the localized spin precessed around this magnetic field. The spin precession generated a modulation of the tunneling current at the resonance frequency [10]. The high frequency part of the tunneling current was separated from the DC part by a high-pass-low-pass network and was detected with a spectrum analyzer. This technique was called electron spin noise STM (ESN-STM). ESN-STM measurements reported so far still lack reproducibility and a good signal-to-noise ratio. In some of the presented measurements, only 0.5% of the spectra show a single data point spike above the noise background [11]. As this approach is based on a stochastic excitation and not on a continuous spin precession, the detection of the small signals is challenging. The main hurdles of this technique are, on the one hand, the low signal-to-noise ratio of the rf currents in the pA/nA range and, on the other hand, the lack of a coherent excitation to maintain spin-precession over the measurement time.

Alternative approaches to study magnetization dynamics in STM were developed in the past few years [12,13]. Recently, Baumann et al. reported on the measurement of an electron spin resonance (ESR) signal in a two-level system: the ground and excited spin state of single paramagnetic Fe atoms using SP-STM [12]. In their approach, an rf electric field was generated by the mixing of a continues radio-wave voltage to the STM junction. The adsorption of the electromagnetic wave by the two-level system promoted spin-flips of the Fe atom from its ground state to the excited state. Because a spin-polarized tip was used, these two spin states display a different tunneling conductance. At resonance, a different average spin population compared to the thermal equilibrium yielded a change in the average conductance, and a supplementary DC tunneling current was detected. With this approach to detect magnetic resonance, the two limitations of ESN-STM concerning the excitation and the detection were lifted.

Here, we propose a different experimental approach to study ferromagnetic resonance with SP-STM (FMR-STM). This approach is inspired by the so-called spin-torque diode experiment [14] developed in 2005 by Tulapurkar et al. The method was based on solid state tunneling junctions, and thus, no lateral resolution was obtained. In analogy to this method, a continuous rf voltage is mixed with the STM junction. This rf voltage generates an rf spin-polarized tunneling current from the magnetic tip that in the next step drives a coherent magnetic precession in a ferromagnetic thin film. This magnetic precession can be detected by measuring a supplementary DC current originating from the homodyne detection principle, as will be discussed in detail below. In order to explore the potential of this method, a suitable sample needs to be chosen: a magnetic vortex. This is a good candidate for two reasons: first, the vortex gyration mode occurs at relatively low frequency in the low GHz range [15,16] and is accessible with our experimental setup. Second, vortices and their precession are well-understood. Vortex dynamics are easy to simulate in order to predict the resonance frequency and the rf current needed for the excitation.

In the following, first the concept of this SP-STM based spin-torque diode experiment is presented. Then, some experimental details about the preparation of magnetic vortices and the experiment are given. Finally, a micromagnetic simulation study in relation to the experiments is presented and compared with the experimental results, illustrating the technical difficulties of this approach.

## 2. Materials and Methods

### 2.1. Principle of FMR-STM

To realize FMR-STM, a magnetic tip is used to spin-polarize the current between the tip and sample. The tunneling conductance is a function of the magnetic configuration of the tip and sample due to the tunneling magnetoresistance (TMR) effect [17]. It depends on the angle θ between the orientations of tip and sample magnetization:

(1) $$G\left(\theta \right)=G_{t}\left[1+P_{s}P_{t}\cos\left(\theta \right)\right],$$

where $G_{t}$ is the spin-averaged tunneling conductance of the tunneling contact and $P_{s}$ and $P_{t}$ the sample and tip spin polarizations, respectively [18,19]. A continuous rf voltage (up to 3 GHz) is mixed with the bias voltage of the STM. This high frequency modulation has the functional form:

(2) $$U=U_{0}+U_{\mathrm{rf}}^{t}\cos\left(\omega_{\mathrm{rf}}t\right),$$

where $U_{\mathrm{rf}}^{t}$ is the amplitude of the rf voltage at the tunneling junction, $U_{0}$ is the DC bias, and $\omega_{\mathrm{rf}}$ is the frequency of the rf signal. If there is a magnetization precession under the STM tip, the angular dependency of the tunneling conductance is expressed as a time dependency:

(3) $$\Delta G\propto G_{t}P_{s}P_{t}\cos\left(\omega_{0}t-\phi \right),$$

where $\omega_{0}$ is the precession frequency and ϕ is the phase difference between the modulation of the bias voltage and the modulation of the conductance. Finally, when assuming a linear I(U) relation, the tunneling current is modulated by the product of these oscillations, leading to:

(4) $$\Delta I_{t}\propto \frac{1}{2}U_{\mathrm{rf}}^{t}G_{t}P_{s}P_{t}\left[\cos\left(\left(\omega_{\mathrm{rf}}+\omega_{0}\right)t-\phi \right)+\cos\left(\left(\omega_{\mathrm{rf}}-\omega_{0}\right)t+\phi \right)\right].$$

If $\omega_{\mathrm{rf}}\neq \omega_{0}$, both cosines are time dependent and well above the cut-off frequency of the transimpedance amplifier. The additional modulation cannot be detected. If the rf signal mixed into the tunneling junction hits the resonance frequency, $\omega_{\mathrm{rf}}=\omega_{0}$, the time dependency of one of the cosines is canceled. This results in an additional DC component in the tunneling current called the homodyne current $I_{h}$ with:

(5) $$I_{h}\propto \frac{1}{2}U_{\mathrm{rf}}^{t}G_{t}P_{s}P_{t}\cos\phi .$$

This can be measured at the output of the transimpedance amplifier and corresponds to the local ferromagnetic resonance signal.

As previously mentioned, the detection of a strong resonance signal requires continuous excitation. Typical measurement times used in this experiment were of the order of few milliseconds. This time scale is well above the relaxation time of magnetic precession phenomena. Therefore, the rf voltage mixed in the tunneling junction generates an oscillating spin-polarized current between the tip and the sample, which can drive a coherent magnetic precession through the spin-transfer torque effect.

We now focus on the excitation of magnetic vortices. A vortex is a magnetic nanostructure that exhibits a curling spin structure in the xy-plane around a central region, called the vortex core, that is typically <10 nm in diameter, where the magnetic moments point out-of plane [20,21,22,23,24]. This structure is stabilized by the minimization of the magnetic stray field energy. Its lowest frequency dynamic mode is called the gyroscopic mode and corresponds to the gyration of the core around its equilibrium position with a frequency set by the material’s parameters and the dimensions of the structure [16].

**Figure 1:**
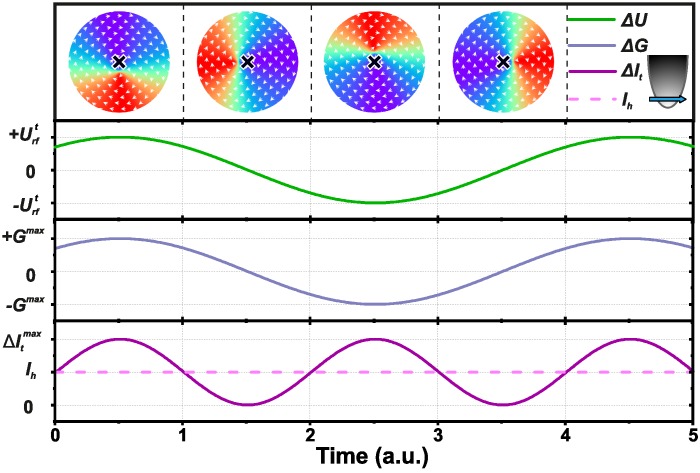
Sketch of the time evolution of the vortex gyration, the rf voltage $U_{\mathrm{rf}}^{t}$, the tunneling conductance, and the resulting supplementary tunneling current excited at resonance and at a phase difference ϕ=0.

Figure 1 presents a sketch of the STM-based spin-torque diode experiment performed on a magnetic vortex. A spin-polarized tip with polarization along the x-direction is positioned above the equilibrium position of the vortex core (black cross at the top of the figure). An rf voltage is applied at the resonance frequency of the gyration mode (green curve). This rf voltage generates an rf component of the tunneling current at the same frequency. The rf current drives the precession of the vortex core through the spin-transfer torque. As a result, the tunneling current oscillates at twice the frequency and shows a homodyne DC-offset current ($I_{h}$, purple curve). This homodyne current is the supplementary DC current (Equation (4)) that can be measured by the transimpedance amplifier:

(6) $$I_{h}=\frac{1}{2}U_{\mathrm{rf}}^{t}G_{t}P_{s}P_{t}A\cos\left(\phi \right),$$

where *A* is the oscillation amplitude of the magnetization under the STM tip (normalized projected magnetization along the tip magnetization direction).

### 2.2. Preparation and Characterization of Magnetic Vortices

**Figure 2:**
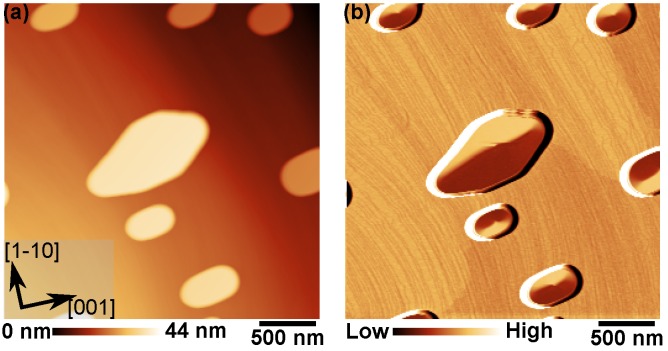
(**a**) STM topography of Fe islands on W(110) recorded at 4.2 K; (**b**) Simultaneously recorded spin-polarized dI/dU map showing magnetic contrast in the islands in the form of magnetic vortices ($I_{t}$ = 1 nA, $U_{0}$ = −400 mV, $U_{\mathrm{mod}}$ = 50 mV).

To realize magnetic vortices, Fe islands prepared on a tungsten surface were used. As reported in the literature, depending on the size, these islands may show a vortex magnetic structure [20,21,22]. A clean W(110) surface was prepared *in situ* under ultra-high vacuum (UHV) by cycles of annealing in O_2_ atmosphere at ≈1200 °C and high temperature annealing to ≈2200 °C. Ten monolayers (ML) of Fe were epitaxially grown on the stepped W(110) surface. A subsequent post-annealing to ≈575 °C for 10 min led to the formation of Fe islands with the aspect ratio necessary to stabilize a magnetic vortex. Fe-coated tips with an in-plane spin polarization were used in SP-STM experiments. As Fe has a high spin polarization ($P_{t}\approx 0.4$), both the spin-torque effect and the homodyne signal (see Equation (5)) were maximized, but the ferromagnetic coating of the tip caused a magnetic stray field of unknown direction and size. Figure 2 shows both a large-scale STM topography and the simultaneously recorded SP-STM differential conductance (dI/dU) map of the surface. Isolated islands on top of a 1 ML-thick Fe wetting layer were formed. These iron islands displayed lateral sizes between 300 nm and 1 μm and heights between 15 nm and 20 nm. The SP-STM dI/dU map reveals a magnetic contrast corresponding to a vortex structure: bright and dark areas correspond to a high and low dI/dU signal, i.e., to a parallel and antiparallel alignment between tip and local sample magnetization, respectively. The magnetization in each of the islands rotates in-plane around the vortex core, which itself points out-of-plane (this was verified using an out-of-plane sensitive tip; experiments not shown here). In the largest island of this image, the structure near the vortex core is slightly deformed. This is due to the tip stray field displacing the vortex core. The restoring force that tends to stabilize the vortex core in its equilibrium position is smaller the larger the island is. This also explains why the deformation is not observed in the smaller islands. In the following, we thus focus on small islands.

### 2.3. Details of the Experimental Detection of FMR Signals

All experiments were performed in a home-built STM operating at 4.2 K. The rf signal was sent through a 50 Ω coaxial cable from the rf generator down to the STM sample stage. A detailed description of the transmission line has been published elsewhere [25]. As explained in the previous sections, the experiment proposed here aims to detect a supplementary DC tunneling current corresponding to a magnetic precession. In order to measure this supplementary signal with a good signal-to-noise ratio, lock-in detection was used. The rf voltage was modulated in amplitude (100%) at low frequency $\omega_{\mathrm{mod}}$, well below the cutoff frequency of the current amplifier such that the additional voltage ΔU on top of $U_{0}$ can be written as:

(7) $$\Delta U\left(t\right)=U_{\mathrm{rf}}^{t}\cos\left(\omega_{\mathrm{rf}}t\right)\left[1+\cos(\omega_{\mathrm{mod}}t)\right].$$

The lock-in amplifier was used to demodulate the supplementary DC current at $\omega_{\mathrm{mod}}$. In order to understand all contributions to the signal that is measured with this method, we write the Taylor expansion of the time-dependent tunneling current $I_{t}$ for the rf modulation ΔU(t) of the bias voltage $U_{0}$:

(8) $$I_{t}\left(U_{0}+\Delta U\left(t\right)\right)=I_{t}\left(U_{0}\right)+{\left.\frac{dI_{t}}{dU}\right|}_{U_{0}}\Delta U\left(t\right)+{\left.\frac{d^{2}I_{t}}{2dU^{2}}\right|}_{U_{0}}\Delta U^{2}\left(t\right)+\ldots$$

At first, we considered the case far away from the resonance ($\omega_{\mathrm{rf}}\ll \omega_{0}$ or $\omega_{\mathrm{rf}}\gg \omega_{0}$). No component of the modulation of the tunneling conductance at $\omega_{0}$ was expected to be detected, and the homodyne current $I_{h}$ vanished (see Equation (5)). Nevertheless, a supplementary signal can be measured. In a tunneling junction, the current-voltage characteristic I(U) is generally non-linear, and the introduced rf signal induces a rectification current [25,26]: the rf voltage generates a high frequency modulation of the tunneling current around its DC value. For a non-linear I(U) characteristic, the average value of this high-frequency modulation caused a DC offset in the tunneling current, which was also detected by the lock-in. Its expression can be calculated by developing the second order term of the Taylor expansion of the tunneling current (Equation (7)) and extracting the Fourier coefficient at $\omega_{\mathrm{mod}}$:

(9) $$I_{\mathrm{nonlinear}}=\frac{1}{2}{U_{\mathrm{rf}}^{t}}^{2}{\left.\frac{d^{2}I_{t}}{dU^{2}}\right|}_{U_{0}}.$$

This expression for the non-linear current $I_{\mathrm{nonlinear}}$ induced by rectification of the non-linear I(U) characteristic is an approximation in the limit of small rf amplitude. The full signal can be easily calculated by considering the expansion up to higher orders. For the sake of simplicity, we restrict ourselves to the leading and lowest order. Note that this signal will come on top of the FMR signal, but does not depend on the rf frequency. It only depends on the local curvature of the I(U) curve and the rf amplitude in the tunneling junction $U_{\mathrm{rf}}^{t}$. Nevertheless, as will be discussed later, this signal can be mistaken for the resonance signal.

Now, we consider the case of resonance ($\omega_{\mathrm{rf}}=\omega_{0}$). In this case, also a homodyne current is expected. Its expression can be calculated by developing the first order term of the Taylor expense of the tunneling current (Equation (7)):

(10) $${\left.\frac{dI_{t}}{dU}\right|}_{U_{0}}\Delta U\left(t\right)={\left.G(t)\right|}_{U_{0}}\Delta U\left(t\right),$$

where ${G\left(t\right)|}_{U_{0}}$ is the time-dependent tunneling conductance at $U_{0}$. Inserting Equation (3) yields:

(11) $${\left.G(t)\right|}_{U_{0}}=G_{t}\left(U_{0}\right)\left[1+P_{S}P_{t}\cos\left(\omega_{0}t-\phi \right)\right].$$

Similarly, the expression for the homodyne current $I_{h}$ can be deduced by calculating the Fourier coefficient of Equation (8) at $\omega_{\mathrm{mod}}$. As before, this expression is an approximation in the limit of a small rf amplitude. Thus, at resonance, the total supplementary current due to both FMR and non-linear effects can be express as:

(12) $$\Delta I=I_{h}+I_{\mathrm{nonlinear}}=\frac{1}{2}U_{\mathrm{rf}}^{t}G_{t}P_{s}P_{t}A\cos\left(\phi \right)+\frac{1}{2}{U_{\mathrm{rf}}^{t}}^{2}{\left.\frac{d^{2}I_{t}}{dU^{2}}\right|}_{U_{0}}$$

During a magnetic resonance experiment, the rf frequency was swept, and the supplementary current was measured. Away from the resonance, only $I_{\mathrm{nonlinear}}$ was detected. At the resonance, the supplementary homodyne current should appear. As previously reported [25,27,28], the transmission of the rf signal to the tunneling junction of an STM was not constant over frequency. When just keeping the rf amplitude of the rf generator constant, the rf voltage at the junction became a function of frequency, and via the non-linear current, a frequency-dependent signal was measured even if there were no spin-dynamics-related effects at all. Moreover, if the cables and connectors for the rf signal contained magnetic materials, the transmission function became a function of applied magnetic field, and the illusion of a resonance signal may arise. Thus, it is imperative to compensate the rf amplitude at the tip $U_{\mathrm{rf}}^{t}$ for transmission effects [25,27,28].

In our experiment, the transmission function has been determined by placing the tip away from the magnetic islands on the Fe wetting layer. Using the method explained in full detail in [25], the non-linear current itself was used to determine the rf amplitude at the tip for every frequency, and compensation was achieved by adjusting the rf amplitude of the generator, accordingly. This way, $U_{\mathrm{rf}}^{t}$ was kept constant during the frequency sweeps.

## 3. Results

### 3.1. Modeling of Vortex Excitation

In order to estimate the resonance frequency of the gyration of a vortex in an individual Fe island, as well as the rf current required to excite this mode, micromagnetic simulations were carried out. Furthermore, such simulations provided information on the amplitude and line shape of the homodyne signal expected in this system. According to Equation (6), the homodyne current depends on the experimental parameters ($U_{\mathrm{rf}}^{t},G_{t},P_{s}$ and $P_{t}$), but also depends on *A*, the actual oscillation amplitude of the magnetization below the STM tip, and ϕ, the phase difference to the excitation. These two last parameters are unknown and will be determined from the micromagnetic simulation. Then, the amplitude of the homodyne signal, as well as its line shape were extracted.

**Figure 3:**
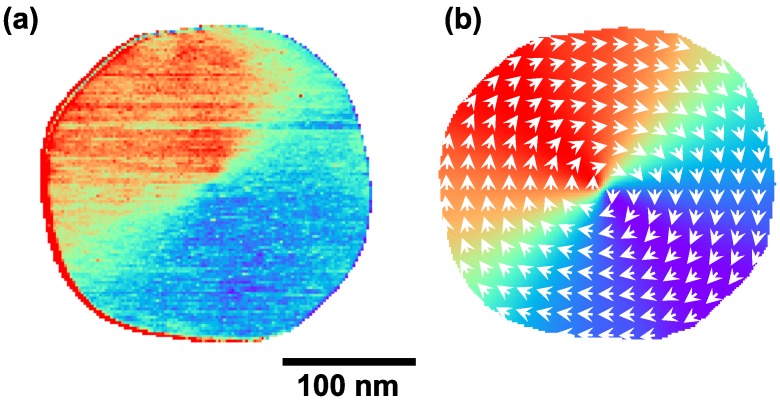
(**a**) Spin-polarized dI/dU map of an Fe island on W(110). $I_{t}=$ 1 nA, $U_{0}=$ −400 mV, $U_{\mathrm{mod}}=$ 50 mV; (**b**) Micromagnetic simulation of an island of the same shape as in the experiment.

Micromagnetic simulations were carried out using the finite element package “nmag: from the University of Southampton [29]. The geometry of the island was determined from high resolution STM topographic images. The 3D shape of the island was approximated by a finite element mesh. The maximum distance between two mesh points was set to 3 nm, i.e., roughly the exchange length in Fe. Bulk parameters for saturation magnetization, damping, and the Fe stiffness constant provided by nmag were used. Further, we used the bulk cubic magnetocrystalline anisotropy constant of Fe, i.e., neglecting surface and interface anisotropies and possible strain-induced anisotropies. The simulations in the following were realized by considering the geometry of an Fe island of an approximately round shape of 250 nm in diameter. Figure 3a presents the experimental spin-polarized conductance map obtained on this island with an in-plane spin-polarized tip. Figure 3b shows the corresponding micromagnetic relaxed vortex magnetic structure obtained with the micromagnetic simulation. Red/blue in the conductance map corresponds to high/low conductance (sample magnetization is parallel/antiparallel to the tip spin polarization). Green corresponds to an intermediate conductance: the sample magnetization perpendicular to the tip spin-polarization. As can be seen from the figure, the experiment and simulated magnetic structure agreed.

**Figure 4:**
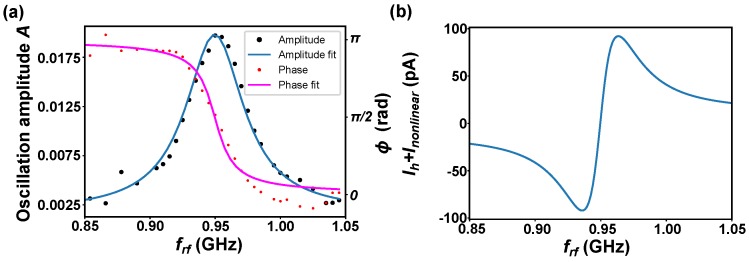
(**a**) Simulated amplitude and phase of magnetization precession *A* at the vortex core as a function of frequency for an excitation rf current of 80 nA; (**b**) Simulated homodyne current.

In order to determine the resonance frequency of the gyration mode in this island, micromagnetic simulations were carried out. An in-plane field applied along the $\left[1\bar{1}0\right]$ direction displaced the core by ≈10 nm from its equilibrium position along the [001] direction. The external field was abruptly turned off, and the time evolution of the magnetization was calculated. The resonance frequency was found to be $f_{0}=960$ MHz. FMR experiments were simulated by modeling the local spin transfer torque, which is the dominant excitation mechanism. The current was injected at the center of the vortex in an area of 1 × 1 nm^2^. We neglected field-like torque, as well as the action of the rf electromagnetic field on the excitation. We varied the excitation frequency $\omega_{\mathrm{rf}}$ in the vicinity of the resonance frequency and calculated the excitation amplitude *A* and the phase difference ϕ at the center of the vortex and varied the excitation current. Figure 4a presents the amplitude *A* (left axis; black dot) as a function of frequency for an excitation rf current of 80 nA. A clear Lorentz-shaped resonance can be observed. The blue line represents a Lorentzian fit to the data. Note that excitation currents in the tens of nA regime can be achieved in SP-STM experiments, and the amplitude corresponds to an angular precession of the magnetization of about 1°. Figure 4b displays the phase difference ϕ (red dots) between oscillation and excitation as a function of frequency for the same excitation current. Below the resonance, both were in phase. At the resonance, they were at −π/2 and above at −π, characteristic for a simple resonance of a harmonic oscillator. Figure 4b displays the expected homodyne current $I_{h}$ (see Equation (6)). At resonance, the signal vanished due to the −π/2 phase. Below and above the resonance frequency, a bipolar signal showed up. The signal was ≈200 pA, which corresponds to 0.08% of the total rf tunneling current. This illustrates the small size of the expected signal under reasonable assumptions for the excitation current achievable in SP-STM. Note that the frequency-dependent variations of the measured supplementary current $I_{h}+I_{\mathrm{nonlinear}}$ in the case that the transmission to the tunneling junction was not taken into account were of the order of several nA. The expected resonance signal was significantly lower than the effects caused by the non-compensated rf amplitude and its non-linear rf current. Thus, it is vital to correct for non-constant transmission.

### 3.2. Experimental Results

**Figure 5:**
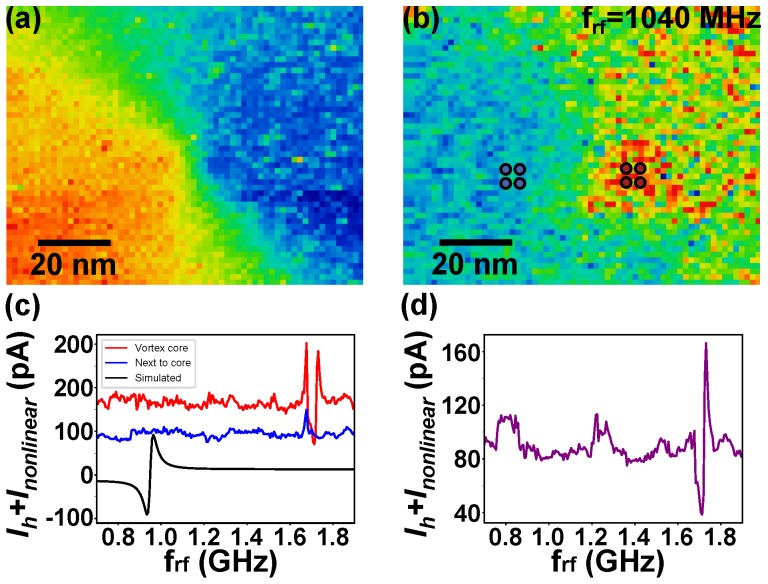
(**a**) dI/dU map of the vortex core area ($I_{t}=$ 10 nA, $U_{0}=$ −400 mV, $U_{\mathrm{mod}}=$ 50 mV) and (**b**) the corresponding map of the supplementary current at frequency $f_{\mathrm{rf}}=$ 1040 MHz ($I_{t}=$ 20 nA, $U_{0}=$ 5 mV, $U_{\mathrm{rf}}^{t}=$ 15 mV); (**c**) Supplementary current as a function of rf frequency measured in two different areas of (**b**) indicated by blue (next to vortex core) and red (vortex core) dots and simulated homodyne current (black); (**d**) Normalized supplementary current on the vortex core.

In the following, we focus on experimental results obtained including correction for transmission for the above island. Figure 5a displays a high resolution map of the spin-polarized dI/dU signal of the vortex core in the center of the island. On the same area, the supplementary current was recorded with compensation for transmission. Thus, here, $U_{\mathrm{rf}}^{t}$ is a constant. The observed map of the supplementary current shown in Figure 5b clearly shows a maximum at the core position. However, this maximum was there for all chosen excitation frequencies between 0.8 and 1.9 GHz. Thus, this maximum cannot be caused by the homodyne current, but is due to the non-linear current. In fact, the I(U) curves (not shown) of the vortex core differed from those recorded outside of the core. This difference in electronic density of states can be due to spin–orbit interaction, magnetostriction (which is also visible in the STM-topography), or the non-collinear magnetoresistance effect [30]. Again, this result highlights the need for cautious interpretation of FMR-STM data. Finally, Figure 5c shows the frequency-dependent supplementary current recorded on the vortex core (red) and next to it (blue). The two curves were offset due to the non-linear current. The spectrum with transmission corrections was much flatter than the uncompensated ones (not shown). Besides statistical noise, the curves show correlated noise, which is likely due to a non-perfect correction of the transmission. Note that the transmission correction itself had an error of about 4% [25]. No statistically-relevant resonance peaks were observed next to the vortex core. There was, however, a clear resonance structure in the spectrum recorded on the vortex core at 1.7 GHz. To illustrate the amplitude of the expected resonance signal, we plotted the simulated curve of the homodyne current using parameters similar to the experimental ones (black curve). In the vicinity of the resonance peak of the calculations, no resonance peak could be found in the experimental curve. The calculated and measured peak intensities were similar, but their frequencies disagreed. In order to further increase the signal-to-noise level and to focus on the difference of the signals recorded on the vortex core and next to the vortex core, we plotted the ratio of the two curves (on the vortex divided by next to the vortex) in Figure 5d. Interestingly, the shape of the normalized spectrum agreed well with that of a ferromagnetic resonance with a resonance at 1.7 GHz. The micromagnetic calculations neglected the stray field of the Fe tip, surface, and interface anisotropies, as well as strain-induced anisotropies. All these could potentially lead to shifts in the resonance frequency. Thus, calculations have to be taken with a grain of salt. The disagreement in resonance frequency was, however, large, so we were not able to identify the observed peak with a ferromagnetic resonance positively.

## 4. Discussion

As the comparison between experimental and simulated homodyne current shows, magnetic vortices are in principle suitable for FMR-STM, but only with extreme rf currents that are difficult to manage. This is due to the height of the islands. On the one hand, they need to be high enough such that the stray fields lead to magnetic vortices. One the other hand, the spin-torque effect is an interface effect, and increasing the height of the islands reduced the oscillation amplitude. The small precession angle of about 1° led to small FMR signals in comparison to the statistical and systematic noise. Instead, non-collinear spin structures in monolayer films, such as magnetic skyrmions [31,32,33], are much more suitable for FMR-STM. Further, theoretical modeling lacks some of the microscopic parameters for vortex gyration such that a positive identification of the gyration mode by its resonance frequency becomes problematic. Furthermore, this is better for magnetic skyrmions, as microscopic parameters can be calculated ab initio and can be verified independently by experiments of the spin structure as a function of magnetic field [33].
